# Clinical Outcomes and Prognostic Factors of Stereotactic Radiotherapy for Spinal Metastases With Epidural Spinal Cord Compression Grades 1–2

**DOI:** 10.1002/cam4.71529

**Published:** 2026-02-23

**Authors:** Hongqing Zhuang, Yao Xiao, Jun Li, Yuxia Wang, Cheng Cheng, Liyuan Tao, Feng Wei, Yaqiang Liu

**Affiliations:** ^1^ Department of Radiation Oncology China‐Japan Friendship Hospital Beijing China; ^2^ Department of Engineering Physics Tsinghua University Beijing China; ^3^ Department of Radiation Oncology Beijing Tsinghua Changgung Hospital Beijing China; ^4^ Medical Examination Centre Peking University Third Hospital Beijing China; ^5^ Orthopedic Department Peking University Third Hospital Beijing China

**Keywords:** epidural spinal cord compression (ESCC), local control efficacy, spinal metastases, stereotactic body radiotherapy (SBRT), toxicity

## Abstract

**Objective:**

Investigate the efficacy of stereotactic radiotherapy in patients with metastatic tumors accompanied by epidural spinal cord compression (ESCC) of Grades 1–2 but without vertebral instability who did not undergo surgery.

**Methods:**

Based on lesion volume and spinal cord compression degree, SBRT doses of 24 Gy/1f, 30 Gy/3f, and 40 Gy/5f were given. The primary endpoint was local progression‐free survival (LPFS). Secondary endpoints included toxicity and overall survival (OS). A comparative analysis of LPFS outcomes among different ESCC grades was conducted, and multivariate analysis was used to assess factors influencing local treatment efficacy.

**Results:**

Among 71 lesions, only three showed local progression. No statistically significant difference in LPFS was observed between ESCC Grades 1 and 2 (*p* = 0.769). Esophagitis/oral mucositis occurred in 15 cases (33.3%), including 1 case of Grade 3 (2.2%). Nausea occurred in 14 cases (19.7%), with 12 cases of Grade 1 (16.9%) and 2 cases of Grade 2 (2.8%). Vomiting occurred in 3 cases (4.2%), with 2 cases of Grade 1 (2.8%) and 1 case of Grade 2 (1.4%). Acute pain occurred in 3 cases (4.2%). One case (1.4%) had late neurological damage of Grade 2, and there were no cases of spinal cord injury. ESCC grading was not a significant factor affecting LPFS in tumor SBRT (*p* = 0.693).

**Conclusion:**

Preliminary findings suggest that for ESCC Grades 1–2 metastatic tumor patients who have no vertebral instability, stereotactic radiotherapy alone has outstanding local control and minimal toxicity. SBRT could be an effective alternative to surgery combined with radiotherapy.

## 1 Introduction

Spinal metastases with spinal cord compression, notably in cancers such as renal, thyroid, breast, and non‐small‐cell lung cancer following treatment [1, 2, 3, 4, 5], are often managed by separation surgery followed by stereotactic body radiotherapy (SBRT) [6, 7, 8, 9]. However, as SBRT gains more ground in clinical practice, promising outcomes have been witnessed in many patients with spinal cord compression treated solely with SBRT [10], albeit lacking solid clinical evidence. Currently, there is a lack of clinical studies investigating definitive stereotactic radiotherapy for patients with spinal metastases presenting as ESCC (Epidural Spinal Cord Compression) Grades 1–2. If patients in this subset (ESCC Grades 1–2) can achieve satisfactory results through direct SBRT without surgery, it would hold significant clinical value by omitting the surgical step in their treatment. This study gathered patients with ESCC Grades 1–2 spinal metastases who underwent direct SBRT to observe clinical efficacy and prognostic factors. It is the first study to provide a curative dose for spinal metastases accompanied by spinal cord compression, aiming to offer insights for spinal metastases clinical management.

## 2 Methods

### 2.1 Study Design and Participants

**FIGURE 1:**
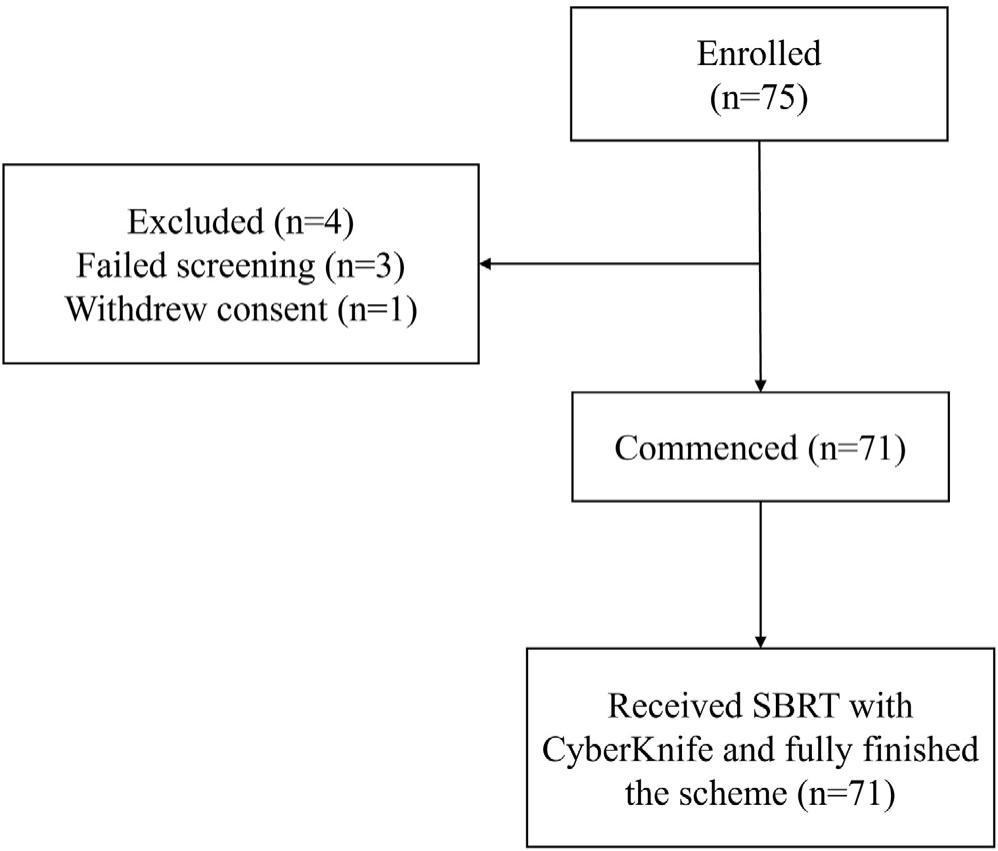
Patient disposition. Flow chart showing the number of patients who were enrolled, commenced treatment, and completed the fully finished SBRT scheme.

This single‐arm prospective study collected data from 65 patients with 71 metastatic lesions classified as ESCC Grades 1–2 (Figure 1). The enrolled patients utilized the ESCC scale to determine the grade of ESCC based on T2‐weighted MR images [11] prospectively. ESCC Grade 1 was defined as a tumor with epidural extension and abutting the spinal cord without displacement. Grade 2 was defined as a tumor that displaces or compresses the spinal cord without circumferential tumor extension or obliteration of the cerebrospinal fluid space. The spine instability neoplastic score (SINS) classification system was employed for spine stability assessment. Exclusion criteria for the study included: patients with severe pain who were unable to lie flat; patients with vertebral instability requiring surgical intervention; and patients with widespread systemic metastases not indicated for stereotactic radiotherapy. The primary endpoint of this study was local control, while secondary endpoints included patient survival and toxicity. Patient enrollment began in September 2018 and was completed by January 2024. The study was approved by the Ethics Committee of Peking University Third Hospital (approval number: M2020259), conducted under committee supervision, and all patients provided informed consent (clinical trail: NCT04192383). Specific details are provided in Table 1.

**TABLE 1:** Patient baseline characteristics.

Characteristic	Value (cases/%)
Cases	71
Gender (cases/%)	
Male	45/63.4
Female	26/36.6
Age (year)	
Range	32–75
Median	60
Lesion site (cases/%)	
Cervical vertebra	19/26.8
Thoracic vertebra	32/45.1
Lumbar vertebra	20/28.2
Disease type (cases/%)	
Lung cancer	11/15.5
Thyroid cancer	7/9.8
Breast cancer	3/4.2
Renal cancer	49/69.0
ESCC grade	
1	28
2	43
SINS score	
0–6	54
7–12	17
Dose (Gy)	
Range	24–40
Median	30
Fraction	
1	6
3	31
5	34

### 2.2 Treatment Methods

All patients underwent SBRT via the CyberKnife platform. The tumor lesions were contoured as the gross tumor volume (GTV), including epidural and paraspinal components of the tumor, with an additional 3 mm margin to form the planning target volume (PTV). The overlapping part between PTV and the spinal cord needed to be retracted. Treatment was administered with real‐time tracking of the spine, and 1–5 fractions of irradiation were given based on the relationship between lesion volume and the spinal cord. Doses ranged from 24 to 40 Gy. Based on the lesion and the degree of spinal cord compression, 95% PTV of 24 Gy/1f, 30 Gy/3f, and 40 Gy/5f were administered. If the lesion involves the vertebral body and occupies < 2/3 of the vertebral body volume, or if it is a small lesion involving the appendages, we give 24 Gy/1f. If it involves the entire vertebral body or the vertebral body and unilateral appendages, we generally give 30 Gy/3f. If it involves the vertebral body and bilateral appendages, we give 40 Gy/5f. In fact, the final decision on the fractionation mode is based on the spinal cord's tolerance limit. The dose calculation uses the Monte Carlo algorithm, the dose line uses the 70%–80% dose line standard, the PTV covers more than 95%, the GTV covers 100%, and the limit for organs at risk refers to the limit standard given by the current AAPM. Treatment was on a daily basis. Further details are provided in Table 1. During stereotactic radiotherapy, patients were treated according to the standard medical treatment plan. For patients currently receiving chemotherapy, stereotactic radiotherapy was performed between chemotherapy cycles.

### 2.3 Follow‐Up and Assessment

The first efficacy assessment was conducted approximately 3 months after the completion of the initial treatment, with subsequent evaluations every 3 months within the first year. After the first year, follow‐up assessments were performed every 3–6 months based on efficacy. Imaging evaluations, including MRI, CT, and bone scans, were employed during follow‐up. Efficacy assessment criteria were based on the Spine Response Assessment in Neuro‐Oncology (SPINO) group's expert consensus on response assessment after stereotactic radiotherapy for spinal metastases, published in 2015 [12].

### 2.4 Statistical Analysis

Statistical analysis was performed using SPSS 26.0 software. The Kaplan–Meier method was employed to construct overall survival (OS) and local progression‐free survival (LPFS) curves. Cox regression analysis was used for multivariate analysis of factors influencing patient treatment efficacy.

## 3 Results

This prospective study collected data from 65 patients with 71 metastatic lesions classified as ESCC Grades 1–2 (Figure 1). The median follow‐up time was 33.7 months. Among these cases, there were 11 with non‐small cell lung cancer (all post‐drug therapy), 7 with thyroid cancer, 49 with renal cancer, and 3 with breast cancer. Of these, 28 patients were classified as ESCC Grade 1, and 43 as ESCC Grade 2. Lesions were distributed in the cervical spine in 19 cases, thoracic spine in 32 cases, and lumbar spine in 20 cases. The majority of lesions had SINS scores ranging from 0 to 6, accounting for 76.1% (54/71). A smaller portion had scores from 7 to 12, constituting 23.9% (17/71), all of which were confirmed to have stable spines after surgical consultation [13].

### 3.1 Local Progression‐Free Survival Between ESCC Grades 1 and 2

**FIGURE 2:**
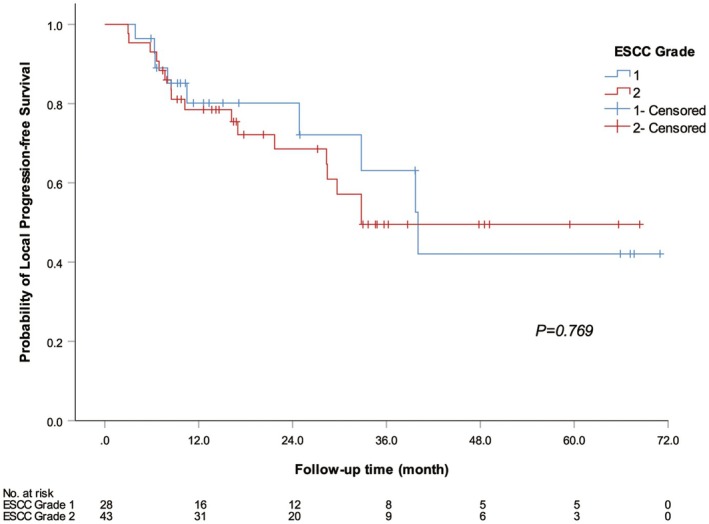
Local progression‐free survival curve for patients with different ESCC grades. The blue curve represents the LPFS curve for patients with ESCC 1 grade. The red curve represents the LPFS survival curve for patients with ESCC 2 grade. There was no significant difference in LPFS between patients with different ESCC grades (*p* = 0.769).

For patients with ESCC Grades 1–2 accompanied by spinal cord compression, direct SBRT demonstrated excellent overall local control. Among the 71 lesions treated with SBRT, only three demonstrated local progression. Of these, one case was of ESCC Grade 1, and two cases were of ESCC Grade 2. There was no statistically significant difference in the median volume for local progression‐free survival between ESCC Grades 1 and 2 (*p* = 0.769) (Figure 2).

### 3.2 Patient Treatment Toxicity

The SBRT‐related toxicity in patients was tolerable during the acute phase, with a low incidence of long‐term toxicity. Among all toxicities in the 71 lesions, there were 45 cases with lesions located at T9 or above. Esophagitis/oral mucositis occurred in 15 cases (33.3%), including 12 cases of Grade 1 (26.7%) and 2 cases of Grade 2 (4.4%), and 1 case of Grade 3 (2.2%). Nausea occurred in 14 cases (19.7%), with 12 cases of Grade 1 (16.9%) and 2 cases of Grade 2 (2.8%). Vomiting occurred in 3 cases (4.2%), with 2 cases of Grade 1 (2.8%) and 1 case of Grade 2 (1.4%). Acute pain occurred in 3 cases (4.2%). One case (1.4%) experienced late neurological damage of Grade 2, exhibiting symptoms and signs of brachial plexus injury, such as restricted movement in both upper limbs. And there were no cases of spinal cord injury. This indicates that the treatment regimen had a relatively acceptable toxicity profile. Specific details are provided in Table 2.

**TABLE 2:** Patient acute and chronic toxicity statistics.

	Grade 1	Grade 2	Grade 3	Total
Esophagitis/oral mucositis (T9 and above)	12	2	1	15
	26.7%	4.4%	2.2%	33.3%
Nausea	12	2	0	14
	16.9%	2.8%	0	19.7%
Vomiting	2	1	0	3
	2.8%	1.4%	0	4.2%
Burst pain				3
				4.2%
Late neurological damage	0	1	0	1
	0	1.4%	0	1.4%
Spinal cord injury				0

### 3.3 Overall Survival and Local Progression‐Free Survival of Patients With ESCC Grades 1–2

**FIGURE 3:**
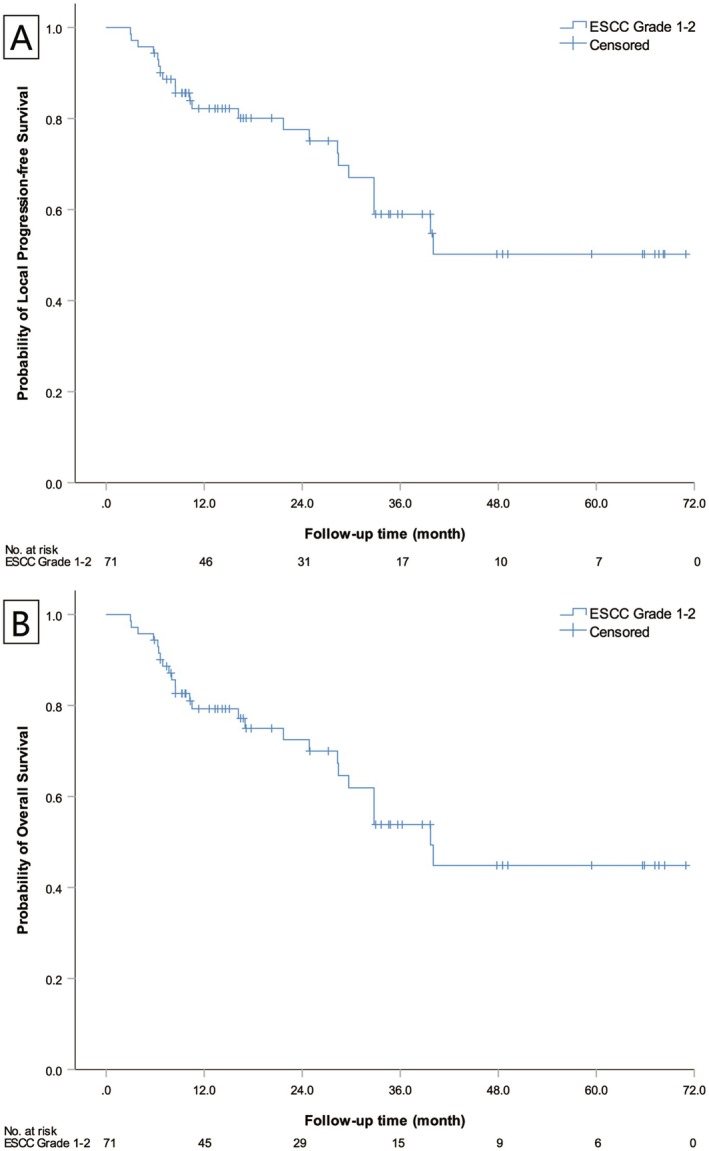
Overall survival and local progression‐free survival curves for ESCC Grades 1–2 patients. (A) represents the LPFS curve for patients with ESCC Grades 1–2. (B) represents the OS curve for patients with ESCC Grades 1–2. The curve trends of the two are close to the same.

Using the Kaplan–Meier method, patient overall survival time and local progression‐free survival time were estimated. The median overall survival time was not reached, and the median local progression‐free survival time was 39.7 months (95% CI 30.1–49.3). The survival curve trends of the two are nearly identical (Figure 3).

### 3.4 Univariate and Multivariate Analysis of Local Progression‐Free Survival

Factors influencing patient efficacy (age, ESCC grading, radiotherapy biological equivalent dose [BED], lesion volume, dose line, ECOG score) were subjected to multivariate analysis. BED was calculated based on an α/β ratio of 10. The multivariate analysis revealed that only the ECOG score was significantly correlated with LPFS (*p* = 0.000). Compared with patients with an ECOG score of 2, patients with an ECOG score of 0–1 had a lower risk of local progression or death than patients with an ECOG score of 2, HR = 0.028 (95% CI 0.006–0.124). ESCC grading was not an independent prognostic factor for LPFS (*p* = 0.693). The statistical results indicated that in patients with ESCC Grades 1–2, as long as the radical dose was administered, the degree of spinal cord compression did not affect the local control efficacy. Specific information is detailed in Table 3.

**TABLE 3:** Results of multivariate analysis for LPFS.

Vairables	Group	Case	Multivariate analysis			
			*B*	*p*	HR	95% CI
Age	< 65	48	0^a^			
	≥ 65	23	0.039	0.092	1.040	0.994–1.088
ECOG score	2	11	0^a^			
	0–1	60	−3.575	0.000	0.028	0.006–0.124
ESCC grading	2	43	0^a^			
	1	28	−0.175	0.693	0.839	0.352–2.001
Lesion volume	≥ 126,830 mm^3^	36	0^a^			
	< 126,830 mm^3^	35	0.517	0.244	1.677	0.703–3.998
Dose line	≥ 76%	37	0^a^			
	< 76%	34	0.261	0.553	1.298	0.549–3.070
BED				0.989		
BED1	81.6 Gy	6	0^a^			
BED2	72 Gy	34	−0.005	0.995	0.995	0.203–4.875
BED3	60 Gy	31	−0.072	0.934	0.931	0.172–5.031

Abbreviation: BED, biological equivalent dose.

^a^ Control group.

## 4 Discussion

The preliminary results of this study indicate that for patients with ESCC Grades 1–2 and metastatic lesions without vertebral instability, choosing stereotactic body radiotherapy (SBRT) alone instead of separation surgery achieves excellent local control with minimal toxicity. This approach is an effective therapeutic option for ESCC Grades 1–2 spinal metastases.

The treatment paradigm of using SBRT for patients with vertebral metastases has evolved with the progress of medical disciplines. Firstly, the rapid development of radiotherapy equipment in the past decade has significantly enhanced the sophistication and precision of SBRT compared to a decade ago [14, 15, 16, 17]. The advancement in radiotherapy equipment and the increased proficiency of clinicians in applying stereotactic techniques have laid a solid foundation for improving the therapeutic efficacy in spinal metastases. Secondly, the evolution of treatment modalities for vertebral metastases across different ESCC grades reflects the shift towards personalized and refined treatment strategies. Historically, the treatment of ESCC across different grades had ambiguous boundaries and discrepancies in disciplinary understanding [18, 19, 20, 21, 22, 23]. However, with the progress of medical disciplines, corresponding changes are observed in the treatment modalities across different grades. The emergence of more personalized treatment approaches for distinct grades indicates progress in the refinement of spinal metastases treatment. Furthermore, the development in understanding spinal radiation tolerance has played a crucial role in enabling the direct application of SBRT for patients with relatively mild compression. Traditionally, conclusions about spinal tolerance were drawn from relatively small sample sizes, and the limits for spinal radiation during spinal cord disease were strict. However, with the accumulation of clinical treatment experience, many cases exceeding the recommended spinal tolerance dose in the literature did not develop radiation‐induced spinal cord disease [18, 24, 25, 26, 27, 28, 29, 30]. As a result, the need for surgical separation as a prerequisite for radiotherapy has decreased for some patients, which is a significant factor in the decision for certain patients to undergo direct SBRT. In summary, the progress in multidisciplinary development and the accumulation of clinical experience have led to a transformative shift in the treatment paradigm for patients with ESCC Grades 1–2.

This study investigates the application of SBRT for patients with ESCC Grades 1–2 and vertebral metastases without instability. Considering the current treatment modalities for spinal metastases and the evolving landscape of radiation therapy, the results suggest favorable clinical outcomes for patients with radiation‐resistant tumors without vertebral instability undergoing direct SBRT. This outcome represents a significant breakthrough in the conventional treatment approach, where patients with metastases associated with spinal cord compression usually undergo separation surgery followed by SBRT. It also provides an efficient way to streamline treatment steps and reduce treatment costs for patients. Moreover, the control rates achieved through SBRT alone for these patients obtained a similar control rate compared to the efficacy of SBRT after separation surgery [18, 31, 32, 33, 34, 35, 36]. Therefore, from a clinical practice perspective, this is a beneficial innovation for patients with spinal metastases associated with spinal cord compression.

However, it should be noted that this study is a phase II clinical trial, and the shift in clinical treatment modalities requires further confirmation through prospective phase III‐controlled studies. We are already initiating the next steps, aiming to further explore the changes in the treatment paradigm for spinal metastases.

In conclusion, this study confirms the efficacy of SBRT for patients with ESCC Grades 1–2 and vertebral metastases without instability and resistance to conventional radiotherapy. It is a valuable exploration for changing the clinical treatment approach for such patients and presents a new direction. Although further confirmation through larger prospective phase III studies is needed, this approach has shown potential clinical value and future trends in the treatment of spinal metastases.

## Author Contributions

**Hongqing Zhuang:** writing – review and editing, investigation, validation, visualization, project administration, resources, supervision, data curation, software, formal analysis. **Yao Xiao:** data curation, software, formal analysis. **Jun Li:** data curation, writing – original draft. **Yuxia Wang:** writing – original draft, methodology. **Cheng Cheng:** methodology, writing – original draft. **Liyuan Tao:** investigation, writing – original draft. **Feng Wei:** methodology, writing – original draft. **Yaqiang Liu:** writing – review and editing, conceptualization, investigation, writing – original draft, methodology, project administration.

## Funding

This research was funded by the Clinical Cohort Construction Program of Peking University Third Hospital, No. BYSYDL2021009.

## Conflicts of Interest

The authors declare no conflicts of interest.

## Data Availability

Data sharing not applicable to this article as no datasets were generated or analyzed during the current study.
